# Deep Learning for Integrated Analysis of Insulin Resistance with Multi-Omics Data

**DOI:** 10.3390/jpm11020128

**Published:** 2021-02-15

**Authors:** Eunchong Huang, Sarah Kim, TaeJin Ahn

**Affiliations:** 1 Department of Advanced Green Energy and Environment, Handong Global University, Pohang-si, Gyeongbuk 37554, Korea; hec1324@gmail.com; 2 Department of Life Science, Handong Global University, Pohang-si, Gyeonbuk 37554, Korea; 22032001@handong.edu

**Keywords:** feature reduction, microbiome, multi-omics, prediction model, feature engineering

## Abstract

Technological advances in next-generation sequencing (NGS) have made it possible to uncover extensive and dynamic alterations in diverse molecular components and biological pathways across healthy and diseased conditions. Large amounts of multi-omics data originating from emerging NGS experiments require feature engineering, which is a crucial step in the process of predictive modeling. The underlying relationship among multi-omics features in terms of insulin resistance is not well understood. In this study, using the multi-omics data of type II diabetes from the Integrative Human Microbiome Project, from 10,783 features, we conducted a data analytic approach to elucidate the relationship between insulin resistance and multi-omics features, including microbiome data. To better explain the impact of microbiome features on insulin classification, we used a developed deep neural network interpretation algorithm for each microbiome feature’s contribution to the discriminative model output in the samples.

## 1. Introduction

Advances in high-throughput DNA sequencing platforms have become essential in the field of gene expression profiling, epigenomics, genomics, and transcriptomics over the past ten years [1,2,3]. The technical developments and decreasing cost of sequencing platforms have made a dramatic contribution to large-scale projects. In particular, human microbiome studies have been accelerated by the advent of next-generation sequencing (NGS) and aim to unravel the association of microbial abundance with health or disease outcomes.

To address the link of humans and their microbiomes to health-related outcomes, the National Institutes of Health (NIH) Human Microbiome Project (HMP) and the second phase [4], the Integrated Human Microbiome Project (iHMP) observed the dynamic alterations in hosts and their microbiomes under particular conditions [5]. The HMP was one of the first large-scale initiative projects to address the linked interactions between hosts and their microbiomes. The first phase project sought to determine whether there were common healthy microbiomes in the absence of overt disease. The ten-year NIH HMP project characterized the microbial communities from numerous body sites and correlated them with phenotypes to determine healthy and disease variations. However, one of the main findings of the HMP was that the microbial composition alone was not correlated with the host phenotype [6,7,8,9]. This finding led to the development of iHMP project, which was designed to gain a more holistic view of host-microbiome interactions over time.

The iHMP project expanded the repertoire of biological properties by providing not only microbial dynamics but also multi-omic analyses, including immunity, metabolism, and dynamic molecular activity, to address the relationship between host and microbiome mechanistically. Within the iHMP project, three sub-projects, comprising pregnancy and preterm birth, inflammatory bowel diseases, and stressors that affect individuals with prediabetes, were included to underpin the mechanisms of human and microbial activity longitudinally. Previous iHMP diabetes-related projects have mainly focused on the time series analysis of insulin sensitivity and resistance in prediabetes patients. The study profiled several molecular patterns, which show that a few markers are sufficient for predicting stress events (i.e., respiratory viral infection versus healthy time points) [10]. Moreover, it also showed that an individual progression of type II diabetes mellitus (T2D) could be predicted before its actual onset via multi-omics analysis. However, the previous study did not address multi-omic factors in classifying insulin sensitivity (IS) and insulin resistance (IR), despite it being feasible.

Now, the emphasis has moved from data generation to effective analysis of data. Substantial challenges are presented, including sample quality control, pre-processing, normalization, and integration of datasets across platforms and techniques. It is worth noting that feature engineering truly reflects the intrinsic relation with the attribute to be predicted, which can significantly affect the performance of any resulting models [11,12]. For instance, typical laboratories facilitating the generation of high-dimensional multi-omic datasets can produce more than 100 gigabytes of information. These high-dimensional multi-omic features may contain noise and misleading features that are detrimental to model performance and may also increase redundant information [13,14]. At the same time, however, the question of obtaining accurate molecular signatures from the biological processes of these complex datasets is complicated. Although classifying IR and IS with a small number of biomarkers is very challenging, we aim to do so by identifying biomarkers that make it possible to distinguish IR from IS.

Considering these converging challenges within the biomedical field, especially with respect to clinical translation, we evaluated whether disease-specific multi-omic variables are present in patients with IR, identified the microbiome-based diagnostic signatures to a classifier setting, and interpreted how selected features contributed to the model output.

## 2. Materials and Methods

### 2.1. iHMP Type 2 Diabetes Mellitus Data Description

The T2D of iHMP was designed to understand the physiological changes that occur in the microbiome and host during viral infection and during changes in glucose levels and IR. This project established a cohort of approximately 60 individuals at risk of diabetes. Under diabetes progression, iHMP T2D performed longitudinal multi-omic analysis to obtain global microbiome-host changes. Specific protocols for each omics profiling and the results of the omics profiling that were analyzed by the Integrative Personal Omics Profiling (iPOP) can be obtained through iPOP Project Data Portal (http://med.stanford.edu/ipop.html).

In this study, we conducted a cross-sectional study of all the samples disregarding the longitudinal profiling of patients. The study population was classified according to the steady state plasma glucose level (SSPG) by iPOP. Subjects with an SSPG greater than 150 mg/dL were classified as IR and below 150 mg/dL were classified as IS. The data consisted of 205 samples classified as IR and 223 samples classified as IS. There were 13,379 RNA transcripts profiled from peripheral blood mononuclear cells, 302 proteins from plasma, 62 cytokines and growth factors from serum, and 51 clinical laboratory features. Out of these features, if any of the features have *NaN* values, missing values, strings, or features that were negative control are excluded. On the other hand, microbiome regularly involves the issue of data containing many zero values. Therefore, we first removed bacterial taxa with a mean relative abundance <0.005%. After filtering, the abundance of each bacterial taxon was normalized with a variance stabilizing arcsine square root transformation [15]. The resulting 29 microbial taxa were used and the total multi-omic features used in this study were 10,783 features.

To develop a model capable of distinguishing IS from IR, subjects were divided randomly in an 8:2 model training dataset to holdout dataset ratio. The holdout dataset was used only to verify the model performance and the training set is randomly divided into 8:2 ratio to obtain training dataset and validation dataset. All the data are scaled to a fixed range by *MinMax* scaling. The overall workflow of this study is demonstrated in Figure S1.

### 2.2. Predictive Models for Insulin Resistance and Insulin Sensitivity (IRIS)

The appropriateness of the reduced features was validated using ensemble classifiers and a deep neural network (DNN) classifier. To obtain optimal model performance, hyperparameter optimization of ensemble models was performed for the learning algorithm using Gridsearch and the best parameters for each model are specified with bold text (Table S1). The code for DNN hyperparameter optimization was built internally to aggregate the best parameters. Similar concept to the Gridsearch, we considered all combinations of hyperparameter of different batch sizes from 15 to 25 in steps of 5, tried a suite of small standard learning rates from 0.0005 to 0.01 and number of nodes was set by dividing by half from the number of features. All the predefined hyperparameters are looped and fitted with the adam algorithm to adjust the learning rate dynamically, sigmoid for calculating predictions, and the remaining layers are activated with the ReLU function. The comparisons of all developed predictive models were based on the area under the receiver operating characteristic (AUC) curve.

### 2.3. Backward Elimination for Feature Selection

Backward elimination is performed to reduce the dimensionality of the multi-omic data and is used for selecting important attributes for classification. The metadata were randomly divided into five folds (index), and in each fold, backward elimination was performed. Briefly, feature importance was obtained from the gradient boosting classifier since the gradient boosting classifier showed the highest AUC compared to other classifiers. Features were arranged as descending order and features with no importance magnitudes were eliminated beforehand. Then, we sequentially erased the features in the feature list and re-calculated the performance drop. Each time, we removed one feature, re-trained the model, and evaluated the performance with the test data. The features of each indexes are eliminated until the AUC of a model drops less than 0.98, and the final features are determined by selecting all the intersecting features across all indexes. The flowchart of backward elimination of this study is demonstrated in Figure S2.

### 2.4. Predictive Models for IRIS with Selected Features

A new predictive model was built and used to re-calculate the model performance to examine the effect of feature reduction. Out of 10,783 features from five different sets of metadata, only 16 features were used to train a new classifier. Hyperparameter tuning of all the five classifiers was performed to optimize the model performance. The best parameters for each model are specified with bold text in Table S1.

### 2.5. Predictive Models for IRIS with Microbiome Feature Substitution

The correlation network was created using the software qgraph version 1.6.5. First, the Pearson correlation of the analytes was obtained using the cor function. Argument use = “pairwise.complete.obs” was used to delete pairwise missing data. Finally, the network was drawn using the qgraph package in combination with graph = “pcor” and the threshold argument (alpha = 0.001) was given to remove edges that were not significant.

Pairwise correlation networks between microbiome features and 16 significant features were calculated, and the corresponding features were replaced with the microbiome features. The final number of features after this experiment was 17 features. Predictive models were rebuilt with 17 features and the model performance was re-calculated to highlight the microbiome data. Hyperparameter tuning of all the five classifiers was performed to optimize the model performance. The best parameters for each model are specified with bold text in Table S1.

### 2.6. Random Sample Permutation

Using the selected 17 features, the DNN model learns randomly split train samples from the training set and generates the test AUC using the holdout dataset. Each number of a permutation sequence uses the randomly split train samples and this is repeated for 100 times. For all the given node values of DNN model in Table S1, DNN builds an optimal model for every permutation with the optimal combinations of learning rate, batch size, and epoch. The histogram for the holdout dataset AUC scores is drawn using matplotlib.pyplot.hist.

### 2.7. Deep Neural Network Interpretation Algorithm

One of the disadvantages of the DNN is the complexity in understanding the precise contribution of a particular feature to the result. One way to address this issue is to input a range of expression values of a feature for a given sample and observe the alterations in the DNN outcome [16]. Specifically, we substituted the value of a feature from its minimum to maximum value across all samples in the dataset and observed the changes in the DNN outcome while the other features were left unchanged. This process was repeated until all features were considered for each sample. The pseudo code for the DNN interpretation algorithm is listed in the Table S4.

### 2.8. Statistical Analysis

Statistical analysis was performed using RStudio (version 3.6.1, http://www.R-project.org). Unless otherwise indicated, the significance tests for the differences between IR and IS within training set and holdout datasets are performed using an independent two-sample *t*-test. Graphical analysis was performed using the GraphPad Prism 8 program (GraphPad Software Inc., San Diego, CA, USA). A *p*-value < 0.05 was considered significant.

## 3. Results

### 3.1. Baseline Characteristics of the iHMP Dataset

The study population consisted of 223 patients with IS and 205 patients with IR. To develop a model capable of distinguishing IS from IR, subjects were divided randomly in an 8:2 model training to holdout dataset ratio. A brief overview of the baseline features of the iHMP dataset is presented in Table 1. According to Table 1, baseline measurements of IR such as SSPG, glucose level, and HbA1c were generally distinguishable from those who were IS. When comparing two datasets, these baseline measurements were distinguishable within each dataset but interestingly, holdout dataset exhibited substantially decreased glucose and HbA1c in IR.

### 3.2. Predictive Models of IRIS with Full Features

**Figure 1:**
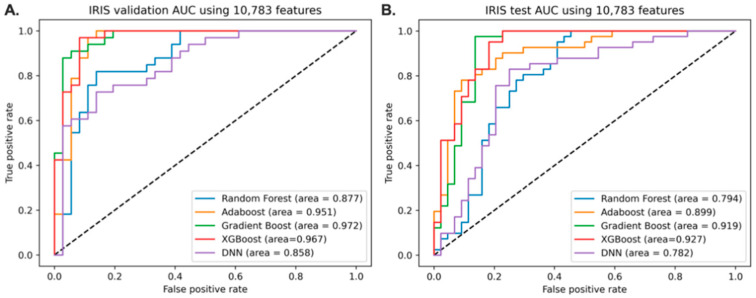
Performance of the models without feature selection. The discriminative models are built using 10,783 features. (**A**). AUC scores of 5 different classifiers using the validation set. (**B**). AUC scores of 5 different classifiers using the holdout dataset. Gradient boosting classifier is selected for showing the highest AUC in validation.

Using all the features from the data, the discriminative models of IR from IS were built using training sets. All features were used to build the discriminative model. The model performances of the five different classifiers are given in Figure 1. The gradient boosting classifier had an AUC of 0.972 in the validation set and 0.919 in the holdout dataset. Because the gradient boosting classifier showed the best AUC in the validation set, the feature importance from the gradient boosting classifier was obtained to construct a better model for IRIS.

### 3.3. Backward Elimination for Feature Reduction and Selection

We performed backward elimination approach to yield minimal number of features contributing to the model. Feature importance magnitudes of the reduced features from the gradient boosting classifier were extracted and arranged from highest to lowest magnitude. Features with no feature importance magnitudes were discarded from the training model, and from the lowest magnitudes, features were erased sequentially and, each time, the alteration of test AUC was observed.

**Figure 2:**
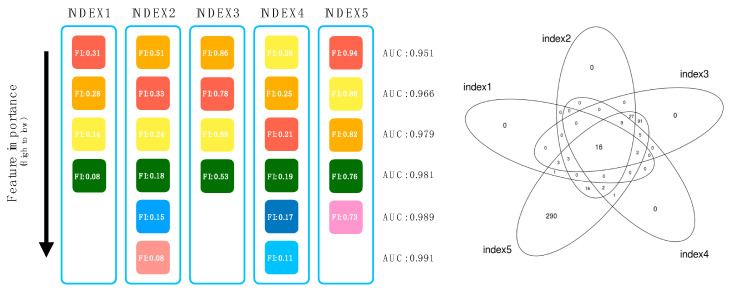
Backward elimination method overview and Venn diagram of features from each index. Schematic diagram of the backward elimination. Feature importance of 10,783 features from gradient boosting was obtained, and from the lowest magnitudes, the features were erased sequentially and each time, the alteration of test AUC was observed. Intersecting features with AUC lower than 0.98 were used for the further research. Venn diagram of the features from each index. In total, 16 features were intersected in all 5 indices.

Features were erased until the test AUC dropped to less than 0.98, and the remaining features were selected for each index. An overview of the method of feature selection is shown in Figure 2. There were 24 features in index 1, 72 features in index 2, 155 features in index 3, 34 features in index 4, and 466 features in index 5. More information about the number of features is shown in Figure 2. From all these indexes, intersecting features were selected as the final features. The statistical analysis of the selected features in both the training and holdout dataset is shown in Table S2.

Of the 10,783 features, 16 features were selected. Among the selected features, six were from clinical measurements (mean corpuscular volume (MCV), EOTAXIN, monocyte absolute value (MONOAB), triglycerides (TGL), creatinine (CR), high density lipoprotein (HDL)), six were from cytokine profiles (stem cell factor (SCF), LEPTIN, granulocyte-macrophage colony-stimulating factor (GMCSF), monocyte chmoattractant protein-1 (MCP1), interleukin 7 (IL7), Fas ligand (FasL)), three were from proteomics (immunoglobin heavy constant mu (IGHM), apolipoprotein E4 (APOE), lysophosphatidic acid (LPA)), one was from the microbiome(genus_*Coprococcus*), and none were from RNAseq.

### 3.4. Predictive Models of IRIS Based on Feature Reduction

**Figure 3:**
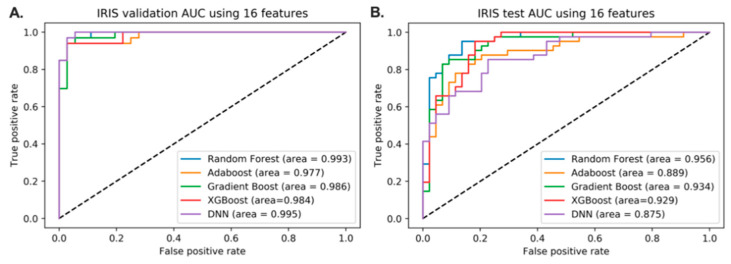
Performance of the models after the feature selection. The discriminative models were built using 16 features. (**A**). AUC scores of 5 different classifiers using the validation set. (**B**). AUC scores of 5 different classifiers using the holdout dataset.

After the features were extracted and selected, the classification step using five different methods was performed on the resulting features. The performance of the models after feature selection is shown in Figure 3. Compared to the predictive models without feature reduction, some models showed improvements in performance. Notably, the test AUC of all the classifiers except Adaboost have increased.

### 3.5. Pairwise Correlation Network between the Microbiome and Extracted Features

IR and IS can be sufficiently classified with 16 features that have already been reduced, but we wanted to discern IR and IS with microbiome data. Within 16 features, only genus_*Coprococcus* remained after backward elimination. Since only one microbial taxon remained, we performed a pairwise correlation network analysis to find any microbial features that can substituted among 16 features.

**Figure 4:**
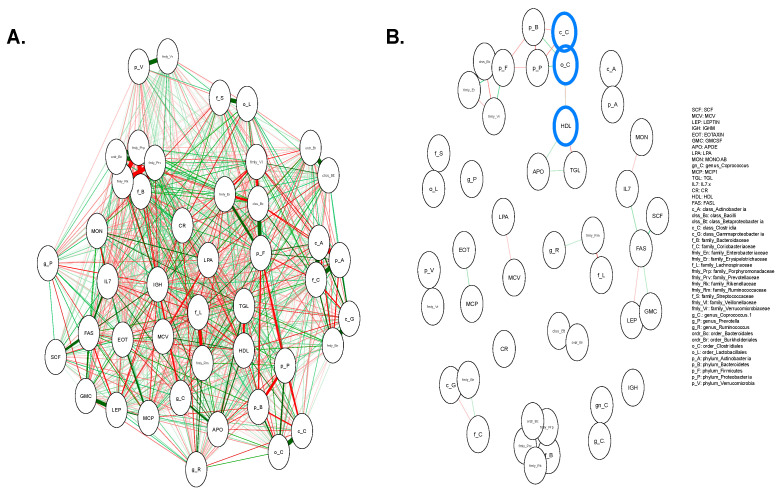
Pairwise correlation network of selected features with microbiome variables. (**A**). Pairwise correlation network of 16 selected features and microbiome variables were plotted without any additional options given. Red lines connected between nodes represent positive correlation and the green lines represent negative correlation. The thickness of the lines indicates a strong correlation between two nodes. (**B**). Pairwise correlation network of 16 selected features and microbiome variables were plotted with threshold (alpha = 0.001). Connected nodes of the selected features and microbiome variables are circled in blue. The red line represents positive correlation, and the green line represents negative correlation between the nodes.

We subsequently constructed a pairwise correlation network over the extracted features and microbiome features to discover the relationships between various biomedical characteristics. The pairwise correlation network of extracted features and the microbiome is shown in Figure 4. Figure 4A depicts the relationship between 16 features that already have been selected via backward elimination with the microbiome variables. Overall, the correlations may interact in complex and unanticipated ways and may provide insights into the potential relations of IS and IR. We applied the threshold (alpha = 0.001) to find the stronger relationships between selected features and microbiome variables (Figure 4B). As a result, HDL was strongly associated with c_C = class_*Clostridia* and o_C = order_*Clostridales*.

### 3.6. Predictive Model of IRIS with the Replacement of Corresponding Features with Microbiome Variables

After applying the pairwise correlation network, we tested whether the model performances were sustainable with the replacement of the extracted features with the microbiome variables. Of the connected nodes between the microbiome variables and the extracted features, the corresponding features were replaced and used to build the discriminative model.

**Figure 5:**
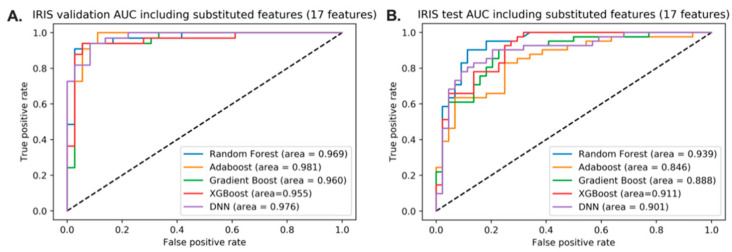
Model performance after substituting the corresponding features with microbiome variables. The discriminative models were built using 17 features. (**A**). AUC scores of 5 different classifiers using the validation set. (**B**). AUC scores of 5 different classifiers using the holdout dataset.

From 16 features, HDL was replaced with two microbiome variables, and a total of 17 features were used to build the discriminative model. The performances of these models are shown in Figure 5. To guarantee that the selected 17 features are well-extracted, random feature permutation was performed (Figure S3). Random feature permutation is repeated 100 times, and each number of permutations use the randomly selected 17 features and generates AUC using validation and holdout dataset. Random feature permutation informs us that the 17 features are better than those picked by randomly and had statistically significant *p*-value.

**Figure 6:**
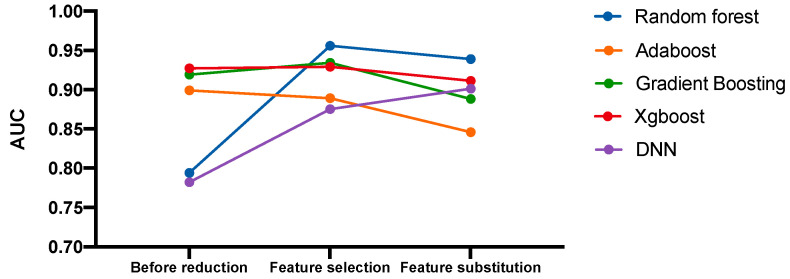
Overall test AUC scores including all previous models. Graphical analysis of model AUC score alteration from no feature reduction to feature substitution. Before reduction = model holdout dataset AUC without any feature reduction (10,783 features); Feature selection = model holdout dataset AUC with feature selection (16 features); Feature substitution = model holdout dataset AUC after substituting corresponding features with microbiome variables (17 features).

As a result, substitution of selected features with microbiome features can sustain model performance (Figure 6). On the other hand, we compared our model result with other T2D models presented by different studies. Even though the characteristics of the data used in different studies were not the same, applied modern learning-machine techniques dealt with the issue of identifying patients with T2D or IR (Table S3).

### 3.7. Acquire the Representative Model with the Highest Frequency in the Random Permutation

**Figure 7:**
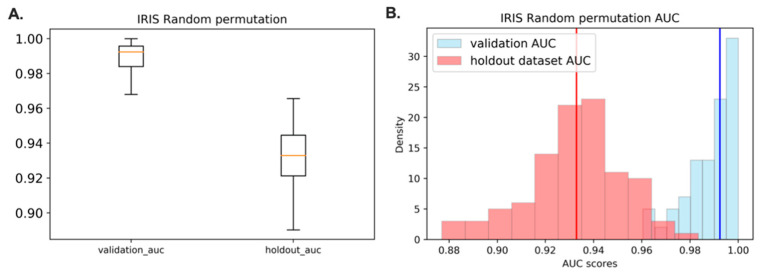
Graphical illustration of DNN model AUC scores after 101 times of random permutation. (**A**). Bar graph illustration of validation and holdout dataset AUC after 101 times of random permutation. (**B**). The histogram shows the distribution of all test AUC scores for every permutation with the optimal combinations of learning rate, batch size, and epoch. The vertical red line represents the median holdout dataset AUC which is at 0.9329 and the vertical blue line represents the median validation AUC which is at 0.9924.

The method proposed by this paper is a fast feature selection for large-scale datasets. It provides proper handling a problem with a selection of suboptimal or diminished features after the classification step. We performed random grouping of samples with the selected 17 features to retrieve distribution of classification performance depending on samples in train data set. Out of machine learning methods we trained, DNN was chosen to be evaluated for this analysis because it showed a consistent increment in AUC across other strategies in the holdout dataset among the training methods compared (Figure 6). The histogram for the test AUC scores of all permutations is shown in Figure 7. The advantage of selecting the central tendency as the final IRIS model identifies as the representative of an entire distribution. DNN model with validation AUC of 0.9924 is a model that represents a case that is not unusual, but that occurs frequently. The representative model with validation AUC of 0.9924 had the AUC score of 0.9440 for the holdout dataset.

### 3.8. Interpretation of 17 Features of DNN Classification Model

We calculated the Shapley value to see how much microbial features have contributed to the IR and IS classification (Figure S4). Class_*Clostridia* and order_*Clostridales* did not affect the classification in the initial model using full features, but when these features were replaced their correlated clinical feature HDL and re-trained the classification model, the two features contributed to the classification model.

**Figure 8:**
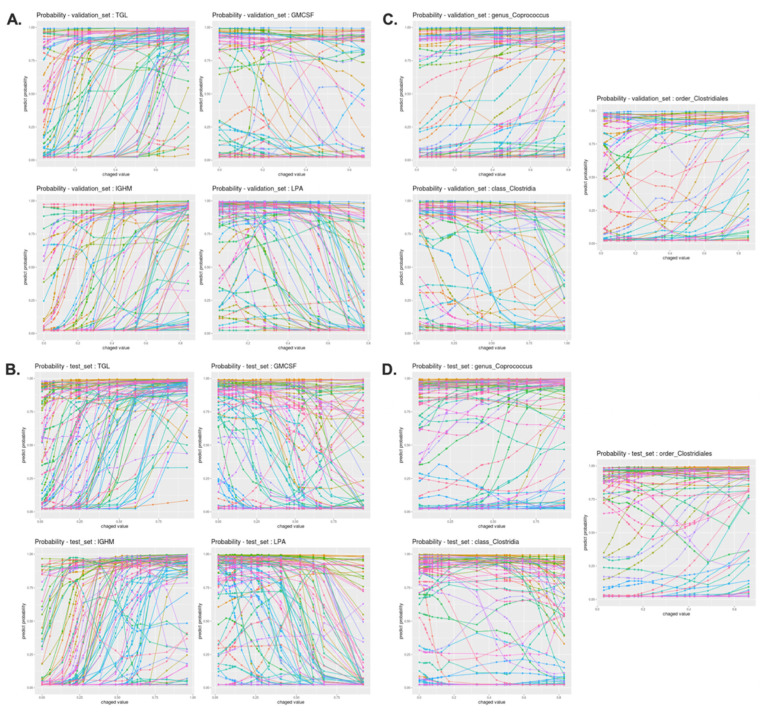
Graphical illustrations of features affecting the DNN classification. Graphical illustration of the contribution of a feature on the outcome of the DNN model. The x-axis represents the range of expression values of a feature and the y-axis represents the predict probability of the DNN model. If the predict probability is close to 1, it indicates that the sample is IR. (**A**). Probability outcome of the top 4 features with highest Shapley values of the DNN model from the validation set, (**B**). Probability outcome of top 4 features with the highest Shapley values of the DNN model from the holdout dataset, (**C**). Probability outcome of the microbiome features from the validation set and (**D**). Probability outcome of the microbiome features from the holdout dataset.

To further understand how 17 features independently contributed to the outcome of DNN model, we applied the interpretation algorithm. Under our experimental settings, the DNN outcome represented a given sample’s probability of being identified as IR. The rationale for this DNN algorithm is inputting a given feature’s range of values and observing the changes in the DNN outcome. Specifically, in this algorithm, we substituted the value of a feature from its minimum to maximum value across all samples in the dataset and observed the changes in the DNN outcome while leaving the other features unchanged. This process was repeated until all features were considered for each sample. We tested both in the validation and holdout datasets of the DNN model trained with 17 features showing that both datasets had similar probability results (Figures S5 and S6, Figure 8).

Overall, this application revealed two important points. First, clinical features that are relevant to insulin such as triglyceride (TGL), lysophosphatidic acid (LPA) and creatinine (CR) showed clear alteration in the DNN prediction suggesting that aberrant level of these clinical features are concordant findings as many other insulin studies. Secondly, this DNN interpretation algorithm clearly emerged microbiome features to classify individuals as IS or IR. Unlike other clinical features, less samples show associated pattern with IS or IR. This suggests that alterations of these microbiome features are potential individualized discriminant biomarkers for IR and IS.

## 4. Discussion

The human microbiome plays an important role in human health, and there is growing evidence that the microbiome can be used as a predictor of various diseases. However, microbiome data pose a huge challenge due to uneven sampling depth, over-dispersion, and zero-inflation under high-dimensional microbiome profiles. Without several steps of careful data engineering, this imbalance induces the data to be highly sparse, which is detrimental to the model performance and may also increase redundant information. A new challenge is presented when the microbiome and other high-dimensional multi-omic datasets are compressed into low-dimensional features. In our study, when the model was generated with reduced features (16 features), the model performance of the five classifiers improved compared to that of the classifiers without feature reduction; however, the feature representation of the microbiome had almost no impact compared to other multi-omic profiles. Except for genus_*Coprococcus*, other microbiome profiles had no influence on the classifier model.

To find meaningful feature representations of the microbiome, we substituted microbiome features with 16 reduced features using a pairwise correlation network. We attempted to substitute for 16 reduced features with more microbiome variables by using a pairwise correlation network with the combination of alpha = 0.01 and alpha = 0.05, to plot the significant edges. With alpha = 0.05, 8 features can be substituted with 16 microbiome variables and with alpha = 0.01, 2 features can be substituted with 5 microbiome variables, but all the classifiers’ test AUCs from alpha = 0.05 and 0.01 dropped lower than 0.75 (data not shown). This does not mean that the dropped features (features with no feature importance magnitudes) are meaningless. We argue that additional features can improve the prediction performance, especially when a well-balanced set of features is augmented.

An interpretation algorithm was applied to evaluate the contribution of a single feature to the outcome of the DNN model. Interpreting the specific contribution of an individual feature is important because the identification of IR driving features in an individual may provide important information for treatment and prognosis. When the values of a feature increase, the probability outcomes of the stem cell factor (SCF) [17], lysophosphatidic acid (LPA) [18], granulocyte-macrophage colony-stimulating factor (GMCSF) [19], interleukin 7 (IL7) [20] and creatinine (CR) [21], and apolipoprotein E (APOE) [22,23,24] clearly switched from IR to IS, as proven by previous studies as having an inverse relationship with insulin resistance. On the other hand, when the values of a feature decrease, the probability outcomes of triglycerides (TGL) [25], monocyte absolute value (MONOAB) [26], and immunoglobulin heavy constant mu (IGHM) [27,28] switch from IS to IR, which have been previously reported to have a direct relationship with insulin resistance. In certain cases, the DNN’s likelihood outcome was barely influenced by changes in the expression value from a single feature. For these samples, DNN was not significantly influenced by a single feature, but by the multiple expression values of the features. In other words, multiple features could adequately classify the samples as IR or IS, but no single feature was able to do so.

More research should be conducted to investigate the MCV, FasL [29], leptin [30,31], eotaxin [32], order_*Clostridales*, class_*Clostridia*, and monocyte chemoattractant protein-1 (MCP-1). Although MCV is irrelevant to IR, a study observed a positive correlation between the diabetes and prediabetes groups [33]. Unclear observations within patients are observed for FasL, leptin, and eotaxin. Although these features are relevant to IR, patient characteristics could have caused different outcomes. Disease progression may vary greatly, which may enhance the implementation of precision medicine at the individual or a sub-population level.

Though variation exists between people’s microbiomes, alteration in the host-microbiota is involved in the progress and development of IR. Several reports observed that genus_*Coprococcus* was enhanced in gestational diabetes mellitus patients [34,35,36]. In general, class_*Clostridia* is reported to have an inverse relationship with IR and were reduced in the diabetic group compared to the control group [37]. On the other hand, one study reported that order_*Clostridiales* was positively related to IS [38], which the result obtained in our study and the study reported previously clearly underline the link between the gut microbiome and IR. Although deeper research must be conducted to elucidate the link between insulin amelioration and human microbiome, but microbiome have the potential to be a good discriminant biomarker for IR.

Lastly, many studies have identified MCP-1 as an insulin-responsive cytokine that promotes IR and glucose intolerance [39,40,41,42]. Contrarily, one of the studies argued that elevated MCP-1 levels in plasma do not influence insulin signaling and have no effect on IR and glucose tolerance in vivo [43]. Based on the probability outcome of MCP-1, a convex shape is observed, meaning that MCP-1 may have markedly different prognoses for IR. Thus, the action of MCP-1 on IR remains unclear, and future studies are necessary to clarify this.

As with any large study, multi-omic datasets appear to be associated with certain variables, but it is not experimentally clear whether such variables are sufficient or informative for their associated disease phenotypes. Moreover, with the continued availability of large samples of multi-omic data, careful consideration must be given for the necessary information not to fade out in downstream prediction.

## Figures and Tables

**Table 1:** Baseline characteristics of the iHMP dataset and the model training and holdout datasets. SSPG = Steady state plasma glucose; GLU = Fasting glucose; HbA1c = Hemoglobin A1c. Data are represented as means ± standard deviation. For statistical analysis, Welch’s unequal variances *t-test* is used to analyze the significance between two groups.

	IS (*n* = 25)			IR (*n* = 32)		*P*
Age	56.144 ± 8.098			57.223 ± 7.062		0.6
BMI	27.474 ± 3.614			29.953 ± 3.558		0.013
Gender	Male (*n* = 11)			Male (*n* = 16)		
	Training set		*p*	Holdout dataset		*p*
	IS (*n* = 179)	IR (*n* = 164)		IS (*n* = 44)	IR (*n* = 41)	
SSPG	101.083 ± 29.354	199.402 ± 35.19	<0.001	105.645 ± 27.412	203.422 ± 31.473	<0.001
GLU	101.994 ± 17.672	92.543 ± 11.954	<0.001	93.386 ± 11.429	89.537 ± 10.46	0.109
HbA1c	5.713 ± 0.423	5.558 ± 0.359	<0.001	5.666 ± 0.557	5.498 ± 0.313	0.088

## Supplementary Materials

The supplementary materials are available online at https://www.mdpi.com/2075-4426/11/2/128/s1. Figure S1: Schematic workflow of this study; Figure S2: Flowchart of backward elimination process. Figure S3: Graphical illustration of DNN model AUC scores after 100 times of random feature permutation; Figure S4: Shapely values of the selected features from the DNN model using holdout dataset; Figure S5: Graphical illustrations of remaining features affecting the DNN classification using holdout dataset; Figure S6: Graphical illustrations of remaining features affecting the DNN classification using validation set, Table S1: Hyperparameter optimization of ensemble models and Deep neural network; Table S2: Statistical analysis of the 16 selected features both in train and holdout dataset; Table S3: Comparison of different machine learning models related to insulin and type 2 diabetes; Table S4: Pseudo code for calculating the contribution of a single feature to the outcome of DNN model.
