# Supplementary material for: Deep Learning for Integrated Analysis of Insulin Resistance with Multi-Omics Data
Source: J Pers Med. 2021 Feb 15;11(2):128. doi: 10.3390/jpm11020128 (PMC7918166; doi:10.3390/jpm11020128)
Supplement: Supplementary file 1 [file jpm-11-00128-s001.pdf]

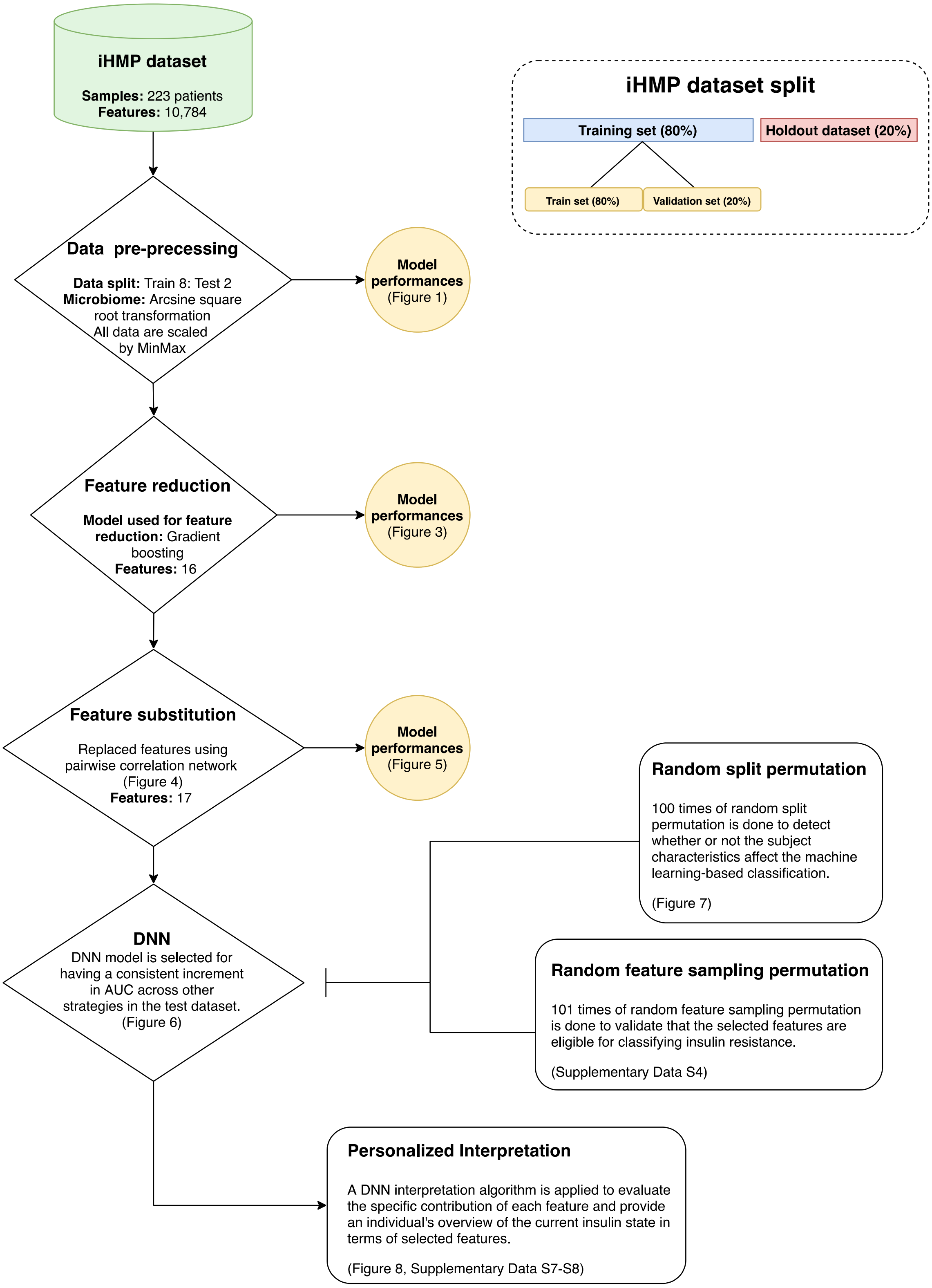

Figure S1. Schematic workflow of this study. Complete workflow for our study. The experimen tal procedure contains 4 major steps: Data preprocessing, feature reduction, feature substitution, a nd personalized interpretation.

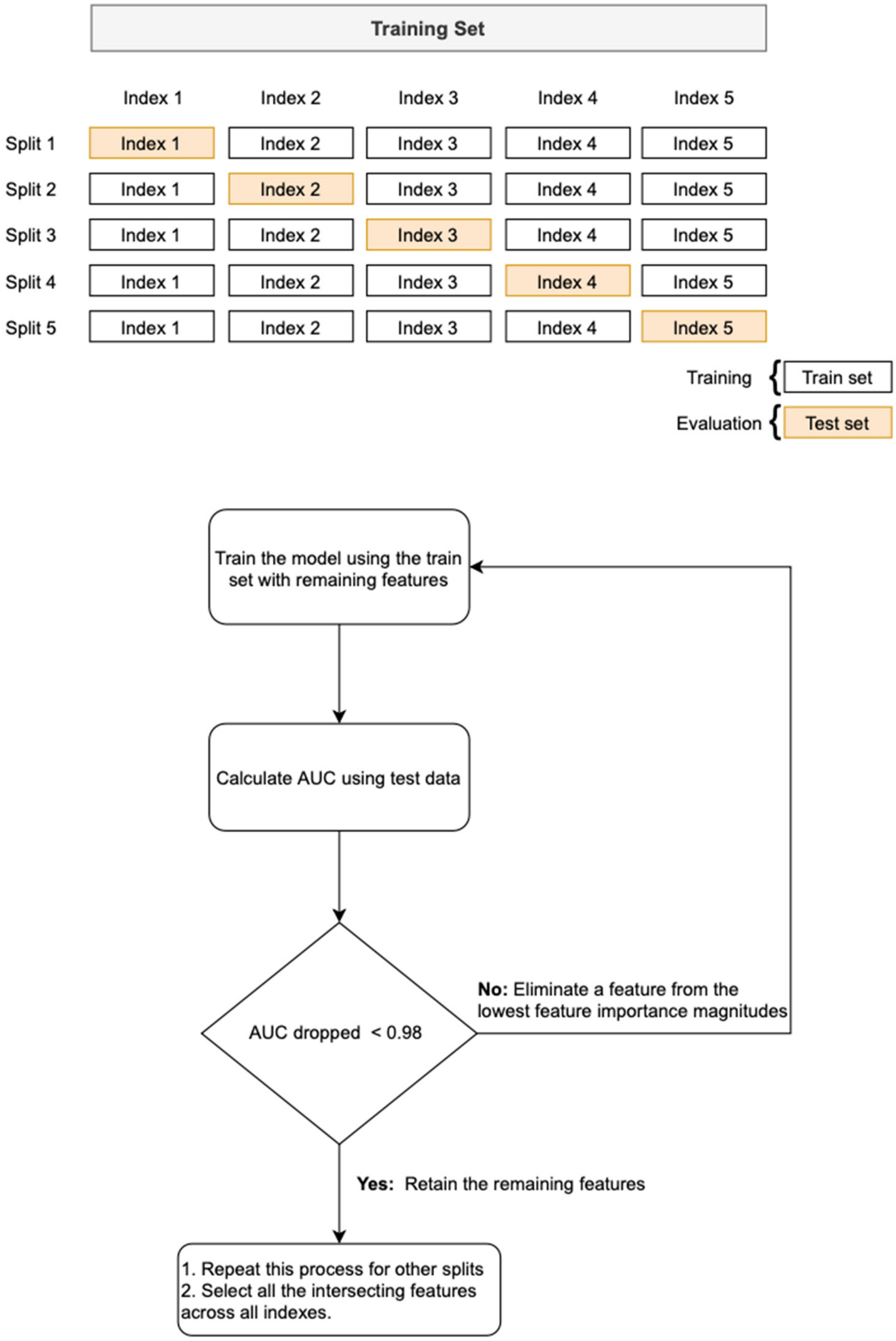

Figure S2. Flowchart of backward elimination process. Flowchart of the backward elimination process. The feature importance of all features is obtained from the gradient boost model. Features are arranged to descending order and each time the alteration of the test data AUC was observed. If the observed A UC did not drop less than 0.98, feature was erased sequentially and re-trained and re-calculated the AU C. This is repeated until the AUC dropped less than 0.98 and done for other indexes.

Model	# of Features	n_estimators	learning_rate	max_depth	min_samples_split
Random Forest	10,783	[50, 100, 150, 200, 250, 300, **350**, 400, 500]		[**5**, 10, 15, 20, 25, 30]	[2, 3, **4**, 5, 6, 7]
Adaboost		[50, 100, 150, 200, **250**, 300, 350, 400, 500]	[0.0001, 0.0005, 0.001, 0.005, 0.01, **0.05**]		
Gradient boosting		[50, 100, 150, 200, 250, 300, **350**, 400, 500]	[0.0001, 0.0005, 0.001, 0.005, 0.01, **0.05**]		[2, 3, 4, **5**, 6, 7]
Xgboost		[50, **100**, 150, 200, 250, 300, 350, 400, 500]	[0.0001, 0.0005, 0.001, 0.005, 0.01, **0.05**]	[**5**, 10, 15, 20, 25, 30]	
Random Forest	16	[50, 100, **150**, 200, 250, 300, 350, 400, 500]		[**5**, 10, 15, 20, 25, 30]	[**2**, 3, 4, 5, 6, 7]
Adaboost		[50, 100, 150, 200, 250, 300, **350**, 400, 500]	[0.0001, 0.0005, 0.001, 0.005, 0.01, **0.05**]		
Gradient boosting		[50, 100, 150, 200, 250, 300, 350, **400**, 500]	[0.0001, 0.0005, 0.001, 0.005, **0.01**, 0.05]		[**2**, 3, 4, 5, 6, 7]
Xgboost		[50, 100, 150, 200, **250**, 300, 350, 400, 500]	[0.0001, 0.0005, 0.001, 0.005, 0.01, **0.05**]	[**5**, 10, 15, 20, 25, 30]	
Random Forest	17	[50, 100, 150, **200**, 250, 300, 350, 400, 500]		[5, 10, 15, **20**, 25, 30]	[2, **3**, 4, 5, 6, 7]
Adaboost		[50, 100, 150, 200, **250**, 300, 350, 400, 500]	[0.0001, 0.0005, 0.001, 0.005, 0.01, **0.05**]		
Gradient boosting		[50, 100, 150, 200, 250, 300, 350, **400**, 500]	[0.0001, 0.0005, 0.001, 0.005, **0.01**, 0.05]		[**2**, 3, 4, 5, 6, 7]
Xgboost		[50, 100, 150, 200, **250**, 300, 350, 400, 500]	[0.0001, 0.0005, 0.001, 0.005, 0.01, **0.05**]	[5, **10**, 15, 20, 25, 30]	

Model	# of Features	learning_rate	batch_size	epoch_list	# of nodes
Deep neural network	10,783	[**0.0005**, 0.001, 0.005, 0.01]	[15, 20, **25**]	[50, 100, **200,** 500]	**[10783, 8626, 6469, 4313, 2156]**
					[10782, 8625, 6498, 4312, 2155]
	16	[**0.0005**, 0.001, 0.005, 0.01]	[15, 20, **25**]	[50, 100, 200, 500]	**[16, 12, 9, 5, 2]**
					[15, 11, 8, 5, 2]
	17	[**0.0005,** 0.001, 0.005, 0.01]	[**15**, 20, 25]	[50, 100, 200, **500**]	**[17, 13, 9, 6, 2]**
					[16, 12, 9, 6, 3]

Table S1. Hyperparameter optimization of ensemble models and Deep neural network. Automatic hyperparameter tuning is used to find the best parameters from the listed hyperparameters. All the predefined hyperparameters are looped and fitted to the estimator on the training set. Fivefold cross validation is given for each set of hyperparameters. Best parameters for each model are specified with bold text. For DNN model, hyperparameter optimization code was built internally.

	Training set		P value	Holdout dataset		P value
	IS (n=179)	IR (n=164)		IS (n=44)	IR (n=41)	
**SCF**	78.426±40.945	64.491±19.425	<0.001	72.413±18.857	64.621±17.222	0.05
**MCV**	89.362±3.461	89.938±5.643	0.261	89.791±3.617	89.912±5.185	0.901
**LEPTIN**	3464.386±3581.2	4364.867±2882.583	0.01	4668.94±3987.608	4518.52±2712.867	0.838
**IGHM**	4.667±0.886	5.259±0.73	<0.001	4.647±0.853	5.179±0.897	0.006
**EOTAXIN**	127.738±71.555	125.494±73.045	0.774	119.761±57.075	110.294±49.491	0.415
**GMCSF**	46.022±40.458	51.138±32.462	0.196	60.048±47.53	54.66±34.63	0.55
**APOE**	4.623±0.626	4.478±0.448	0.013	4.381±0.712	4.498±0.456	0.365
**LPA**	0.501±2.178	-1.159±2.167	<0.001	-0.172±2.238	-1.453±2.461	0.014
**MONOAB**	0.433±0.17	0.453±0.101	0.186	0.462±0.165	0.436±0.121	0.402
**genus_Coprococcus**	0.006±0.013	0.004±0.006	0.014	0.006±0.007	0.006±0.011	0.876
**MCP-1**	655.494±249.907	572.166±312.027	0.007	616.688±219.422	471.194±246.093	0.005
**TGL**	93.006±46.595	125.787±56.626	<0.001	81.795±38.841	139.488±69.181	<0.001
**IL7**	110.682±79.13	73.771±34.103	<0.001	85.474±52.79	83.242±32.78	0.814
**CR**	0.984±0.133	0.844±0.183	<0.001	0.955±0.157	0.9±0.217	0.18
**HDL**	65.179±16.152	52.585±13.836	<0.001	65.432±15.432	50.293±14.294	<0.001
**FASL**	32.386±34.094	28.72±9.163	0.168	28.078±19.069	33.614±26.671	0.278

Table S2. Statistical analysis of the 16 selected features both in train and holdout dataset. SCF = Stem cell factor; MCV = mean corpuscular volume; IGHM = Immunoglobin heavy constant mu; GMCSF = granulocyte-macrophage colony-stimulating factor; APOE = apolipoprotein E4; LPA = lysophosphatidic acid; MONOAB = monocytes absolute value; MCP-1 = Monocyte chemoattractant protein-1; TGL = triglycerides; IL7 = interleukin 7; CR = creatinine; HDL = high density lipoprotein; FASL = FAS ligand. Data are represented as means with standard deviation and analyzed with two sample t-test on vectors of data.

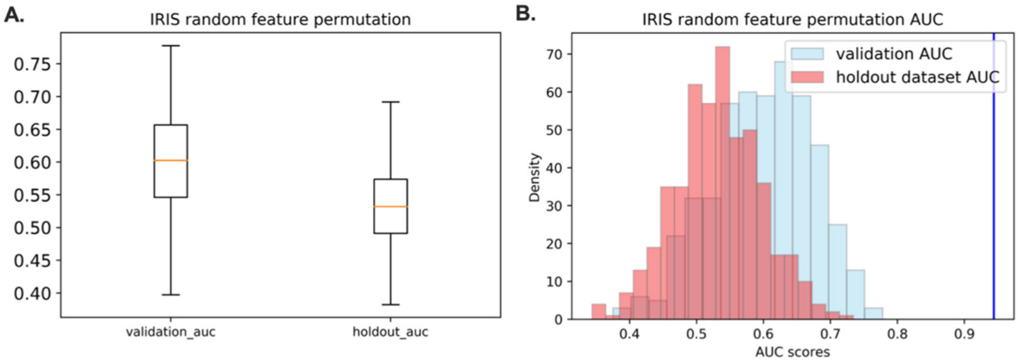

Figure S3. Graphical illustration of DNN model AUC scores after 100 times of random feature permutation. Train_test_split was fixed and from 10,783 features, each number of a permutation sequence uses the randomly selected 17 features and generates the test AUC using the formerly divided holdout dataset. This permutation is repeated for 100 times and each time hyperparameters are tunned to obtain the optimal validation AUCs after each number of a permutation sequence. (A). Bar graph illustration of validation and holdout dataset AUC after 100 times of random feature permutation. (B). The histogram shows the distribution of all test AUC scores for every permutation with the optimal combinations of learning rate, batch size and epoch. The vertical blue line represents the optimal model with the AUC score of 0.9440 for the holdout dataset.

Model	AUC	Study Population	Type of Data	Reference
CRONICAS HOMA-IR	0.686	3,120	Sociodemographical, Clinical	Rodrigo M. et al. 2018 [1]
HOMA-TG index	0.706	224	Anthropometric, Clinical	Khan et al. 2019 [2]
Fasting plasma glucose	0.690			
FIRI	0.674			
HOMAIR	0.632			
HOMA2 index	0.608			
Serum Insulin	0.595			
Quantitative Insulin Sensitivity Check Index (QUICKI)	0.449			
Glucose Insulin Ratio (G/I Ratio)	0.462			
Gradinet Boosting Machine (GBM)	0.847	13,309	Demographical, Clinical	Lai et al. 2019[3]
Logistic regression	0.840			
Random Forest	0.834			
Decision Tree (Rpart)	0.782			
**IRIS (DNN)**	**0.944**	**428**	**Multi-omic data**	-

Table S3. Comparison of different machine learning models related to insulin and type 2 diabetes. Studies that compare more than five machine learning algorithms in different datasets. The aim of these studies is to conduct a systemic review of the applications of machine learning techniques in the field of type 2 diabetes and insulin resistance research with respect to prediction and diagnosis and complications. The highest AUC is shown with bold font.

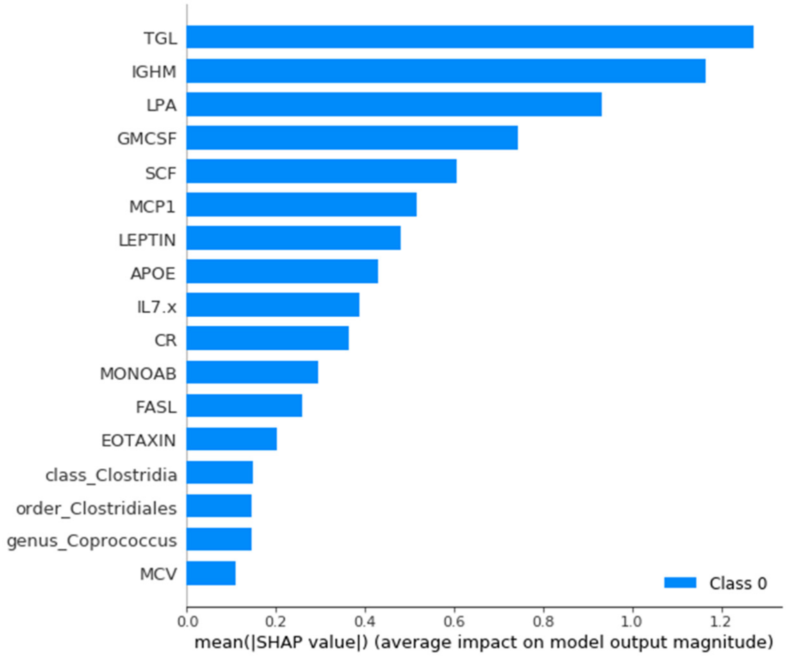

Figure S4. Shapely values of the selected features from the DNN model using holdout dataset. Graphical illustration of Shapely value (SHAP value) outcome of the DNN model using the holdout dataset. The x-axis represents the average impact on the DNN model output, and the y-axis represents the features used in the DNN model. The features are arranged from the highest SHAP value to lowest SHAP value.

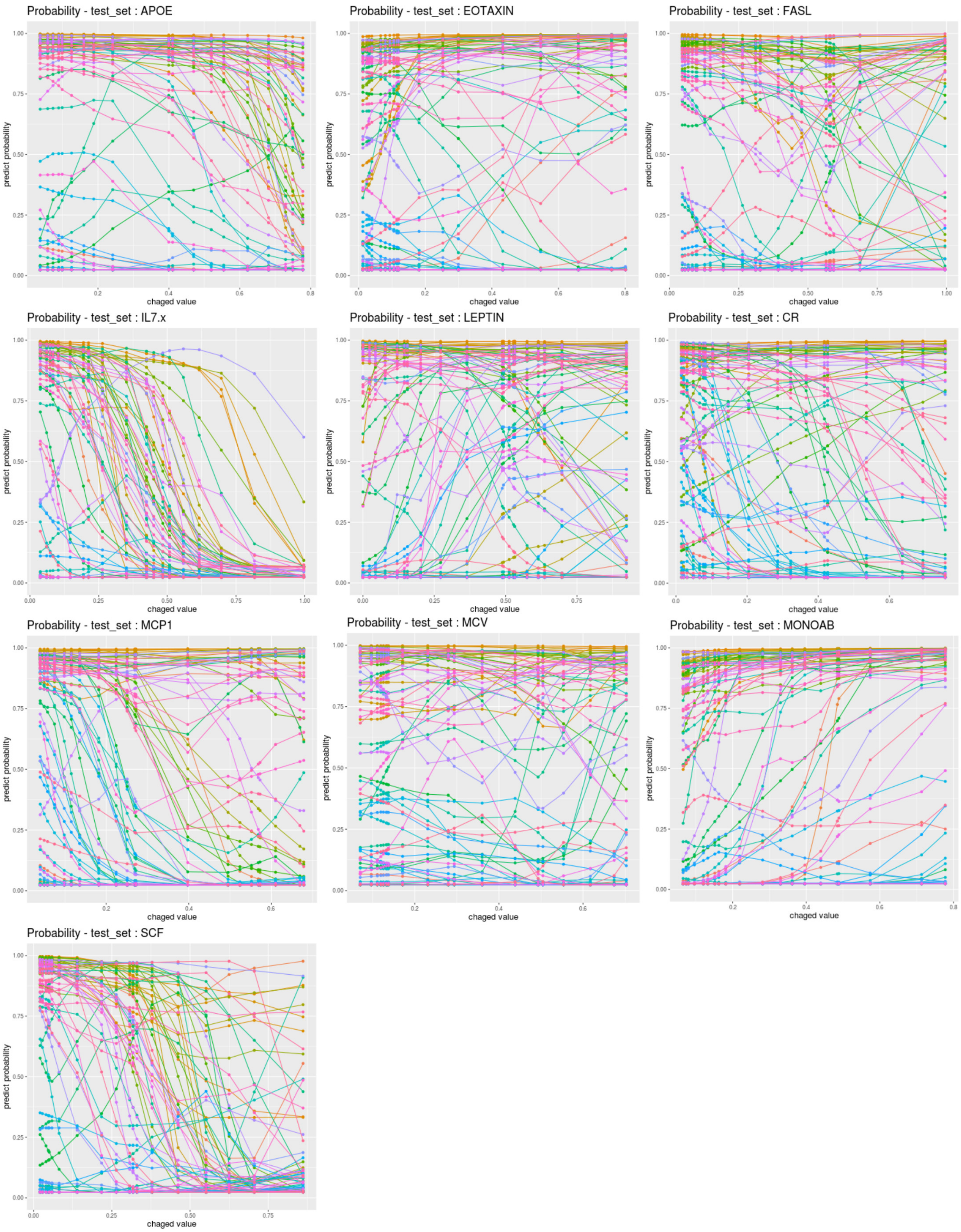

Figure S5. Graphical illustrations of remaining features affecting the DNN classification using holdout dataset. Graphical illustration of a feature to the outcome of the DNN model. The x-axis represents the range of expression values of a feature and the y-axis represents the predict probability of the DNN model. If the predict probability is close to 1, it indicates that the sample is IR.

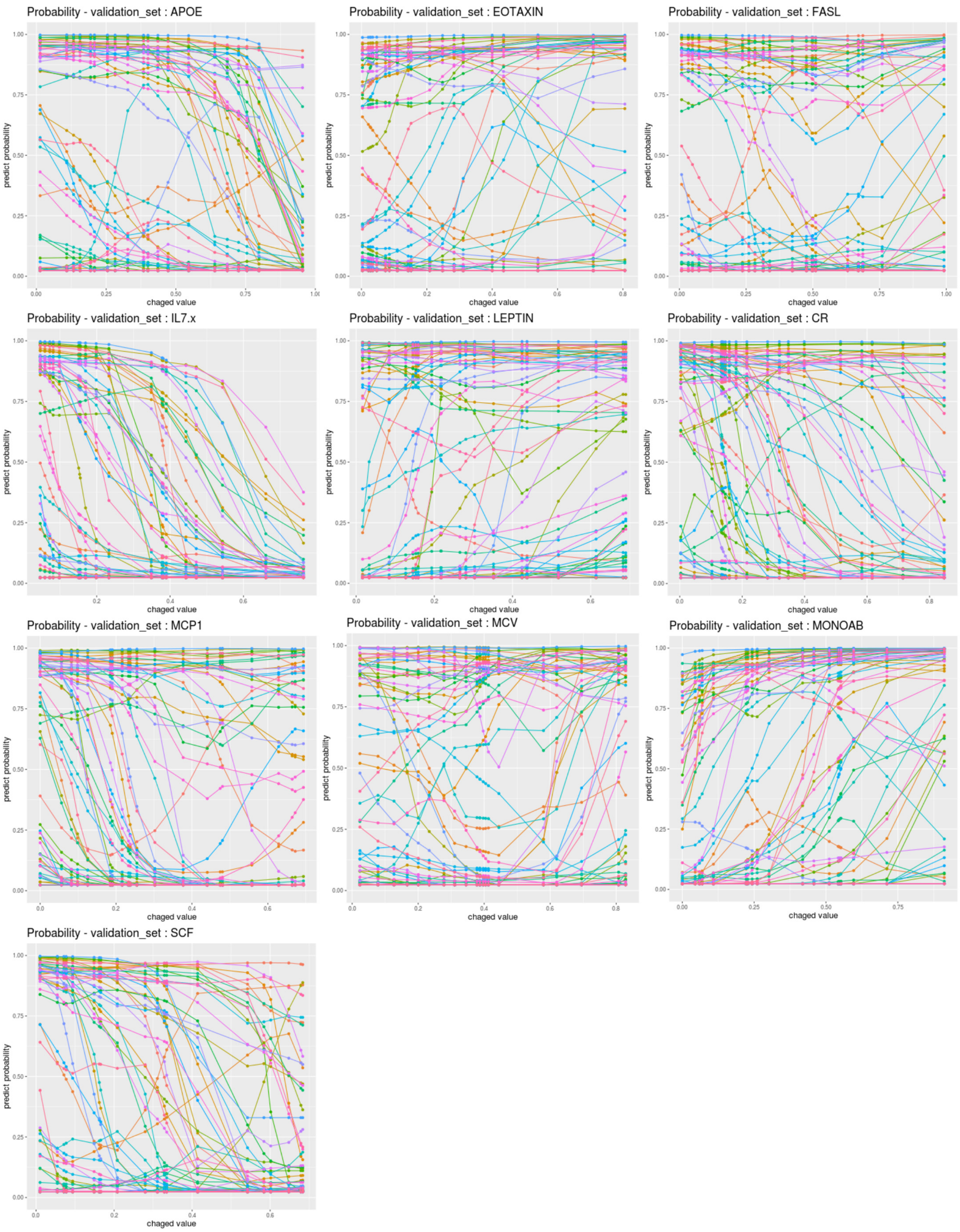

Figure S6. Graphical illustrations of remaining features affecting the DNN classification using validation set. Graphical illustration of a feature to the outcome of the DNN model. The x-axis represents the range of expression values of a feature and the y-axis represents the predict probability of the DNN model. If the predict probability is close to 1, it indicates that the sample is IR.

Algorithm: Calculating the contribution of a single feature to the outcome of DNN model

Input: $\mathbf{X} \in \mathbb{R}^{\boldsymbol{n\,x\,f}}$, matrix of *n* samples and *f* features from the test dataset
The *MinMax Scaling* is implemented in the input
Output: Y, DNN model probability prediction values

Loop feature in feature list:
Obtain feature_value = np.percentile(feature, list(range(0,105,5)))
Loop value in feature_value:
Create new data matrix Z that contains values from the feature_value
Put Z in the trained DNN and get prediction *p*
End loop
End loop

Return *Y*

Table S4. Pseudo code for calculating the contribution of a single feature to the outcome of DNN model. Pseudo code for DNN interpretation algorithm. ß

References

1. Carrillo-Larco, R. M., Miranda, J. J., Gilman, R. H., Checkley, W., Smeeth, L., Bernabe-Ortiz, A., & Group, C. C. The HOMA-IR Performance to Identify New Diabetes Cases by Degree of Urbanization and Altitude in Peru: The CRONICAS Cohort Study. *Journal of Diabetes Research*, **2018**, 1-8. doi:10.1155/2018/7434918
2. Khan, S. H., Khan, A. N., Chaudhry, N., Anwar, R., Fazal, N., & Tariq, M. Comparison of various steady state surrogate insulin resistance indices in diagnosing metabolic syndrome. *Diabetology & Metabolic Syndrome*, 2019, 11(1). doi:10.1186/s13098-019-0439-5
3. Lai, H., Huang, H., Keshavjee, K., Guergachi, A., & Gao, X. Predictive models for diabetes mellitus using machine learning techniques. *BMC Endocrine Disorders*, 2019, 19(1). doi:10.1186/s12902-019-0436-6
